# Identification of African Swine Fever Virus Transcription within Peripheral Blood Mononuclear Cells of Acutely Infected Pigs

**DOI:** 10.3390/v13112333

**Published:** 2021-11-22

**Authors:** Ann Sofie Olesen, Miyako Kodama, Louise Lohse, Francesc Accensi, Thomas Bruun Rasmussen, Christina M. Lazov, Morten T. Limborg, M. Thomas P. Gilbert, Anette Bøtner, Graham J. Belsham

**Affiliations:** 1 Section of Veterinary Clinical Microbiology, Department of Veterinary and Animal Sciences, University of Copenhagen, 1870 Frederiksberg, Denmark; asjo@ssi.dk (A.S.O.); cml@sund.ku.dk (C.M.L.); aneb@sund.ku.dk (A.B.); 2 Section for Veterinary Virology, Department of Virus & Microbiological Special Diagnostics, Statens Serum Institut, 2300 Copenhagen, Denmark; lolo@ssi.dk (L.L.); tbru@ssi.dk (T.B.R.); 3 Center for Evolutionary Hologenomics, The Globe Institute, University of Copenhagen, 1350 Copenhagen, Denmark; miyako.kodama@sund.ku.dk (M.K.); morten.limborg@sund.ku.dk (M.T.L.); tgilbert@sund.ku.dk (M.T.P.G.); 4 Centre de Recerca en Sanitat Animal (IRTA-CReSA), Campus de la Universitat Autònoma de Barcelona, 08193 Bellaterra, Spain; francesc.accensi@uab.cat; 5 Departament de Sanitat i d’Anatomia Animals, Facultat de Veterinària, Universitat Autònoma de Barcelona, 08193 Bellaterra, Spain

**Keywords:** African swine fever virus, transcriptomics, gene expression, PBMCs, RNAseq

## Abstract

African swine fever virus (ASFV) has become widespread in Europe, Asia and elsewhere, thereby causing extensive economic losses. The viral genome includes nearly 200 genes, but their expression within infected pigs has not been well characterized previously. In this study, four pigs were infected with a genotype II strain (ASFV POL/2015/Podlaskie); blood samples were collected before inoculation and at both 3 and 6 days later. During this period, a range of clinical signs of infection became apparent in the pigs. From the blood, peripheral blood mononuclear cells (PBMCs) were isolated. The transcription of the ASFV genes was determined using RNAseq on poly(A)+ mRNAs isolated from these cells. Only very low levels of virus transcription were detected in the PBMCs at 3 days post-inoculation (dpi) but, at 6 dpi, extensive transcription was apparent. This was co-incident with a large increase in the level of ASFV DNA within these cells. The pattern of the virus gene expression was very reproducible between the individual pigs. Many highly expressed genes have undefined roles. Surprisingly, some genes with key roles in virus replication were expressed at only low levels. As the functions of individual genes are identified, information about their expression becomes important for understanding their contribution to virus biology.

## 1. Introduction

African swine fever virus (ASFV) is the sole member of the *Asfarviridae* family. The virus has a large, linear dsDNA genome (ca. 170–190 kbp, depending on the strain) that includes nearly 200 genes (reviewed in [1]). This virus infects domestic pigs together with a range of wildlife species (family *Suidae*), including bush pigs and warthogs in Africa, while wild boar are important hosts in Europe and Asia [2]. In addition, ASFV can replicate within soft ticks (genus *Ornithodoros*) and is unique in being the only known DNA arbovirus. A sylvatic cycle involving replication in soft ticks and warthogs occurs in Africa [2]; the infection is largely asymptomatic in the warthogs but becomes apparent when domestic pigs become involved. Outside of Africa, the transmission of the virus is believed to occur mainly by the direct or indirect contact between infected pigs, generally without the involvement of soft ticks; however, some aspects of its transmission are poorly understood [3].

Many (at least 24) different genotypes of the virus exist in Africa; these are distinguished based on the sequence of the VP72 gene [4,5,6,7,8]. In 1957 and 1960, excursions of a genotype I ASFV from Africa into Europe (Portugal) occurred, and the virus (e.g., the Ba71 strain) was present in the Iberian peninsula until the 1990s [9]. In 2007, a genotype II virus entered into Georgia (in the Caucasus region), and, subsequently, African swine fever (ASF) has become widespread within neighboring countries, such as Russia and those in Eastern Europe. It has also spread into Western Europe, including Belgium and, during 2020, Germany [10]. Furthermore, in 2018, essentially the same virus was reported from China, the world’s largest pig producer [10,11], and quickly moved into many countries in the vicinity (e.g., Vietnam, Korea and Cambodia) and to the Philippines. In 2021, the virus has been introduced into pigs in the Dominican Republic and Haiti [10]; thus, this virus is a global concern.

Infection with ASFV can result in very high levels of case fatality and, thus, has major economic importance. There are no commercially available approved vaccines or antiviral agents to control the disease, so the control measures rely on the culling of infected animals, restrictions on animal movement and high biosecurity [12,13].

Highly virulent isolates of ASFV often cause a peracute to acute disease progression with high fever (>41 °C) and a range of clinical signs, including anorexia and lethargy, which occur within a few days of infection [14,15,16]. ASFV replicates within the cytoplasm of infected cells and encodes its own RNA polymerase and transcription factors. Genes can be expressed at different stages of the virus life cycle, e.g., early (prior to DNA replication) or late (following DNA replication). The open reading frames (ORFs) are closely spaced on the viral genome and are transcribed from the two different strands of the DNA [17,18]. The mRNA transcripts are capped at their 5′-termini and are post-transcriptionally modified at their 3′-termini to generate a poly(A) tail by a virus encoded capping enzyme and poly(A) polymerase, respectively (reviewed in [1]). An initial analysis of the virus gene expression, using the total RNA extracted from the whole blood of pigs infected with the ASFV Georgia 2007/1 virus, has been performed [19] but showed very variable levels of gene expression between animals. Furthermore, a detailed analysis of the ASFV transcription has been undertaken using the cell culture adapted Ba71V strain (genotype I) of ASFV within Vero cells (derived from the African green monkey kidney) [18]. Analyses of the transcription within porcine peripheral blood macrophages and porcine pulmonary alveolar macrophages (PPAM), infected in vitro with isolates of ASFV, have also been reported previously [20,21].

In the pig, the initial sites of virus replication during a natural infection include the pharyngeal tonsils, and the secondary sites include the spleen, lymph nodes and liver (reviewed in [1]). More specifically, the virus primarily replicates in the cells of the monocyte macrophage lineage [22]. In a virus-infected animal, it is not possible to achieve the synchronous infection of all the cells within the animal (c.f. all the cells within a flask). However, in order to follow the time course of infection, in this study, we have examined the expression of virus genes from within the peripheral blood mononuclear cell (PBMC) population (including lymphocytes, monocytes and macrophages) harvested from individual animals at 3 and 6 days post-inoculation (dpi) with ASFV/POL/2015/Podlaskie. This has allowed the transcription of the ASFV genes to be assessed within key target cells of the natural host animal during the progression of individual pigs from being uninfected to being diseased. As the functions of the ASFV genes become known, then information about their expression should assist in the understanding of their contribution to virus biology.

## 2. Materials and Methods

### 2.1. Pigs

Four male pigs, eight weeks of age, were included in this study. The pigs were obtained from a conventional Spanish swine herd (Landrace × Large White). On arrival at the research facility, one week before the start of the experiment, all pigs were found to be healthy by veterinary inspection. Water and a commercial diet for weaned pigs were provided ad libitum.

Animal care and maintenance, experimental procedures and euthanasia were conducted in accordance with EU legislation on animal experimentation (EU Directive 2010/63/EU). The experiment was performed within high containment facilities at the Centre de Recerca en Sanitat Animal (IRTA-CReSA, Barcelona, Spain).

### 2.2. Challenge Virus

For the experimental infection, ASFV was isolated from spleen material obtained from a dead wild boar in 2015 in the Podlaskie voivodeship (province), Poland, as previously described [16]. This virus is designated here as ASFV POL/2015/Podlaskie, the genome has been sequenced [23] and it is very closely related to the updated ASFV Georgia_2007/1 sequence (GenBank Accession no. FR682468.2, [24]). Briefly, clarified spleen suspension was passaged twice in PPAM and the titer of the second passage was then determined by end-point titration in PPAM [16]. For intranasal inoculation of pigs, the second passage virus was diluted in phosphate buffered saline (PBS) to a final concentration of 4 log_10_ 50% tissue culture infectious doses (TCID_50_) per 2 mL, as used previously [16]. Back titration of the inoculum was carried out in PPAM to confirm the administered dose.

### 2.3. Study Design

Upon arrival at the research facility, the four pigs were housed together in a high containment stable unit (BSL-3). After an acclimatization period of one week, the pigs were inoculated intranasally with 2 mL virus suspension containing 4 log_10_ TCID_50_ of the ASFV POL/2015/Podlaskie (see [16]). The time course of the infection in the pigs was followed from their clinical signs, rectal temperatures and using laboratory analyses as described in the following sections.

### 2.4. Clinical Examination and Euthanasia

Clinical scores and rectal temperatures were recorded from individual pigs on each day. A total clinical score was calculated per day based on a modified system from that described previously [16], omitting food intake as it was available ad libitum; also, the rectal temperatures were recorded separately and were not used as part of the clinical score. The total clinical scores were calculated as the sum of scores given in eight categories (see Table 1). This allowed a maximum total clinical score of 31.

The pigs were euthanized after they reached the humane end-points set in the study, which occurred at 6 days post-infection (dpi), by intravascular injection of Pentobarbital following deep anesthesia.

### 2.5. Sampling from the Inoculated Pigs

EDTA-stabilized blood (EDTA blood) samples were collected prior to inoculation at 0 dpi, at 3 dpi and at 6 dpi, just prior to euthanasia. EDTA blood samples were processed directly for isolation of PBMCs (see below).

### 2.6. PBMC Isolation and Processing

Using fresh EDTA blood samples (4 mL) from each pig, PBMCs were isolated using the Histopaque^®^ system (Sigma-Aldrich, St. Louis, MO, USA). The PBMC fraction samples were lysed by addition of Trizol^TM^ Reagent (ThermoFisher Scientific, Waltham, MA, USA), and the samples were stored frozen at −80 °C until further processing.

### 2.7. RNA Purification

Total RNA was extracted from the PBMCs in Trizol^TM^ Reagent, (ThermoFisher Scientific) using the Direct-zol^TM^ RNA MiniPrep kit (Zymo Research, Irvine, CA, USA). This purification system includes a DNAse I digestion to remove host and viral DNA. Analysis of the RNA transcripts was performed using poly(A)+ selected mRNAs. These samples include both viral and host mRNAs, but the selection removes most ribosomal RNA, which were then sequenced (following reverse transcription using random primers, second strand synthesis, adaptor ligation and PCR-amplification) by BGI Europe Genome Center (Copenhagen, Denmark) (termed RNA-T on DNBseq with ca. 40 million reads per sample).

### 2.8. ASFV DNA Detection by Quantitative Real-Time Polymerase Chain Reaction (qPCR)

Following chloroform-mediated phase separation of the remaining volume of PBMCs lysed in Trizol^TM^ Reagent (ThermoFisher Scientific), DNA purification was performed on the interphase material using the MagNA Pure 96 system (Roche, Basel, Switzerland). The presence of ASFV DNA was determined by qPCR assays employing 45 cycles [16,25]. Results are presented as viral genome copy numbers/per mL EDTA blood calculated by reference to a standard curve based on a 10-fold dilution series of a pVP72 plasmid (prepared from a cloned PCR product amplified with primers dCCCGGTCCGAAGCGCGCTTTCCCGGG*ATGGCATCAGGAGGAGCTTTTTG* and dCGAAAGCGGCCGCGGGATCGACTAGTCTA*TTAGGTACTGTAACGCAGCAC*; the sequences in italics match exactly to the ASFV p72 coding sequence from GenBank Accession no. MH681419.1) using as the template DNA extracted from a spleen sample of a pig infected with ASFV POL/2015/Podlaskie [16]).

### 2.9. Data Analysis

#### Mapping of Sequence Reads to the Pig Genome and to the ASFV Genome

Sequence reads (27–47 million per sample) were initially mapped using STAR v. 2.7.0 [26] to the USMARCv1.0 pig genome assembly (Accession no. PRJNA392765), which was derived from a male pig within a population that was approximately one-half Landrace, one-quarter Duroc and one-quarter Yorkshire (see [27]). The analysis of changes in expression of the pig genes within these infected pigs will be reported separately. The unmapped reads were then mapped to the updated ASFV Georgia_2007/1 genome (GenBank Accession no. FR682468.2) using BWA v.0.7.10 [28]; note that one correction to the annotation for the D205R gene was made (using the coding sequence as nt 138482 to 139234). Reads were mapped to individual virus genes using featureCounts, which is a part of the Subread package, v.2.0.3 [29]. The counts were then standardized, when indicated, to take account of the library size and the length of each gene from each sample (gene length corrected trimmed mean of M-values (GeTMM)) [30].

The reads that did not map to the pig genome were also examined using Kaiju [31] to determine the origin of the reads. The parameters used were: minimum match length (11), minimum match score (75), allowed mismatches (5) and maximum *E*-value (0.01).

## 3. Results

### 3.1. Course of Infection in the Inoculated Pigs

**Figure 1:**
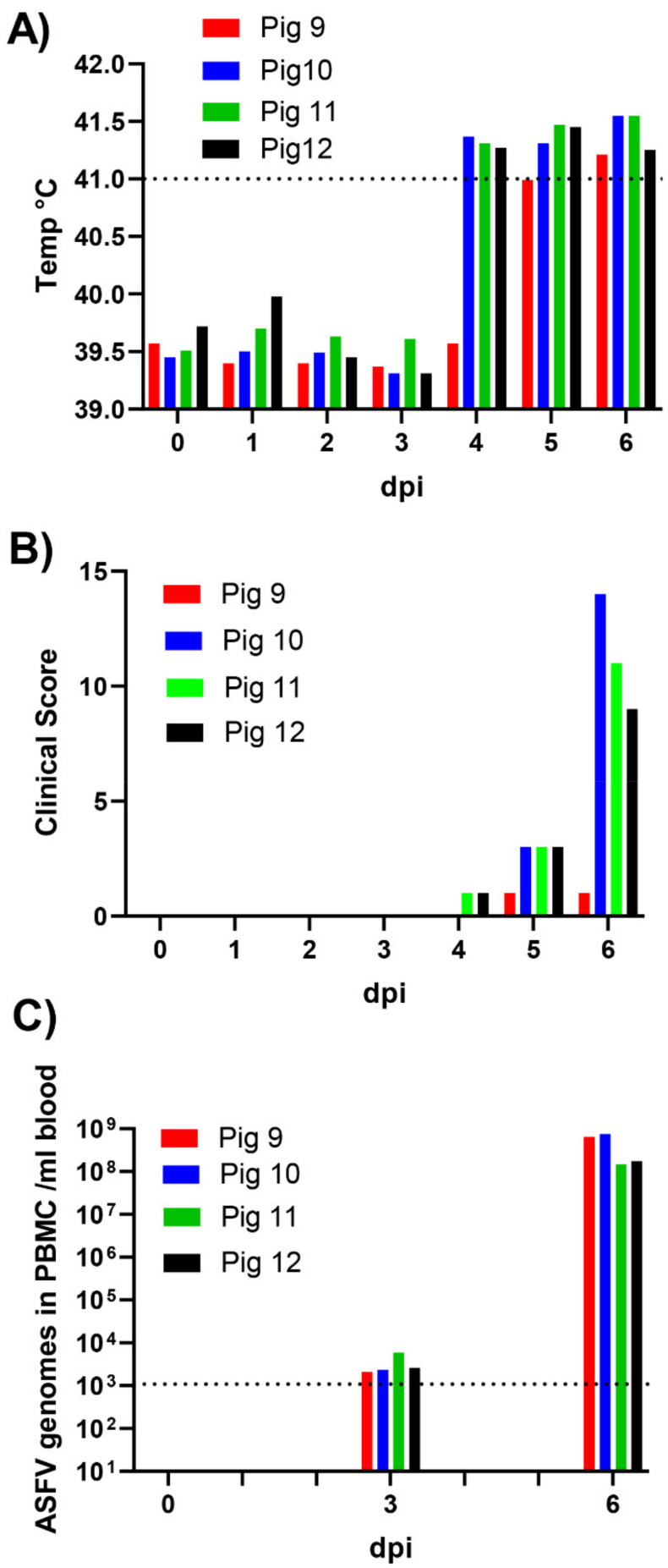
Time course of infection of pigs with ASFV. Pigs were inoculated intranasally with ASFV/POL/2015/Podlaskie at 0 dpi, and then their rectal temperatures were taken on a daily basis (**A**) and clinical signs of disease (for 8 parameters) were also scored (**B**). Blood samples were collected at 0 dpi (prior to inoculation), at 3 dpi and at 6 dpi; the animals were euthanized at 6 dpi. PBMCs were purified from the blood samples (4 mL). The presence of ASFV DNA in the PBMCs (**C**) was quantified by qPCR and values converted to gene copy numbers/mL blood by reference to a standard curve. Levels below 10^3^ ASFV genomes/mL (indicated by dashed line) were outside of the linear range of the assay.

Following intranasal inoculation on day 0, three out of the four pigs (numbers 10, 11 and 12), at 4 dpi, had high fever (rectal temperature above 41 °C; see Figure 1A). Furthermore, at 5 and 6 dpi, all four pigs had high fever. Clinical signs of infection also became apparent and included depression, anorexia, mildly labored breathing, hyperemia of the skin and cyanosis on the ears and distal limbs, plus blood in feces (pig 10). At 6 dpi, all the inoculated pigs were euthanized since pigs 10, 11 and 12 had reached the pre-determined humane endpoint and the remaining animal, pig 9, was euthanized to avoid having a solitary animal. The clinical scores for the four inoculated pigs through the course of the infection are depicted in Figure 1B. All four pigs showed at least some clinical signs of disease, in addition to the elevated temperature by 5 dpi, which had become much more apparent in pigs 10, 11 and 12 at 6 dpi, just prior to euthanasia.

### 3.2. Virus Derived RNA Transcript Analysis from PBMCs of ASFV-Inoculated Pigs

Blood samples (12 in total) were collected separately from the four pigs on day 0 (prior to inoculation), at 3 dpi and 6 dpi (on the day of euthanasia). The PBMC fraction was isolated from the EDTA blood samples, and the level of ASFV DNA in the nucleic acids isolated from the aliquots of the PBMCs was determined by qPCR (Figure 1C). Only low levels of ASFV DNA were detectable in the PBMCs at 3 dpi, but much higher levels (>1000-fold) were present at 6 dpi in all four pigs (Figure 1C). The total RNA was also extracted from the PBMC samples and freed of most of the DNA from the host and the virus. The poly(A)+ RNA (mRNAs) was then selected, reverse transcribed and sequenced (as described in Materials and Methods). The reads (ca. 27–47 million per sample) were initially mapped to the pig genome (resulting in 81–92% of the reads being uniquely mapped, see Table 2), and the unmapped reads were then mapped to the ASFV Georgia_2007/1 sequence (see Table 2). As expected, no reads mapping to the ASFV genome were present in any of the samples collected on day 0, prior to inoculation. At 3 dpi, the PBMCs from pigs 10, 11 and 12 each only generated between 600–850 reads (less than 0.0025% of the total reads) mapping to the ASFV genome, with no ASFV-derived reads detected in pig 9 (Table 2). However, in contrast, at 6 dpi, between 871,681 and 1,945,879 reads (ca. 2–4% of the total reads) from the PBMCs mapped to the ASFV genome from each of the four pigs (Table 2); the lowest numbers of ASFV reads were found in pigs 9 and 10. For pigs 11 and 12, about 40% of the reads that did not map to the pig genome did map to the ASFV genome. Thus, in parallel with the appearance of clinical signs between 3 and 6 dpi and the large increase in the levels of ASFV DNA in the PBMCs of the inoculated pigs (see Figure 1), a huge increase (>1000-fold) in the level of ASFV RNA transcripts within the PBMCs was apparent during this time period.

### 3.3. Transcription of Individual ASFV Genes

**Figure 2:**
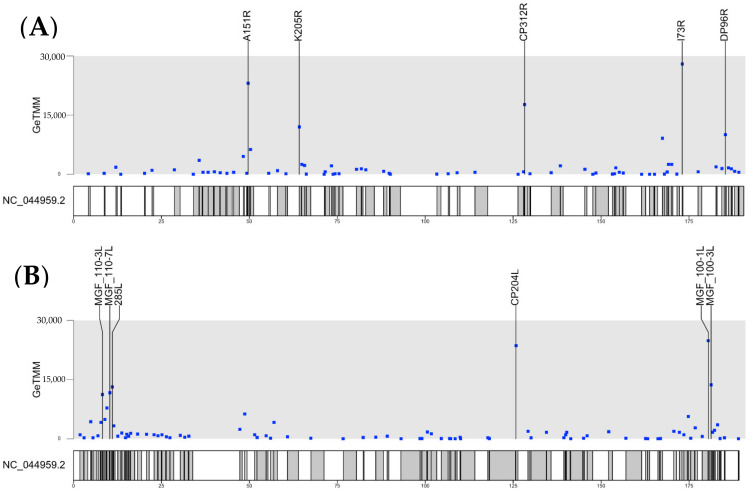
Analysis of ASFV gene transcription within PBMCs of ASFV-infected pigs at 6 dpi. The number of ASFV mRNA derived reads from PBMCs of infected pigs collected at 6 dpi were standardized according to the size of the library and the length of the ORFs (for ORFs >180 nt) and plotted (as GeTMM values) along the entire ASFV genome for the two separate strands of the ASFV genome (panel (**A**), genes transcribed from left to right and panel (**B**), genes transcribed from right to left). The ASFV genes with the highest GeTMM values are indicated.

In general, there was a very good agreement between the pattern of expression of the individual ASFV genes obtained for the PBMCs from each of the different pigs, although the actual levels varied to some extent (see Supplementary Table S1). In particular, the highest levels of virus gene expression were observed in pigs 11 and 12, and the levels of ASFV transcripts present in these cells from pigs 9 and 10 were, on average, about 50% lower. The most highly expressed genes were C312R, CP204L, MGF 100-1L and A151R (see Table 3), while some genes were expressed at a much lower level, i.e., less than 1% of these most highly expressed genes (see Table 4 and Supplementary Table S1). Interestingly, all the identified ASFV coding genes, with ORFs > 180 nt in length, were expressed to some extent (see Figure 2 and Supplementary Tables S1 and S2). Note that there are some very short genes, designated pNG1-7, that were recently identified [18] from the Ba71V genome that were not specifically included here, but several of them correspond to features annotated in the ASFV Georgia_2007/1 genome as ASFV G ACD 00xx0 (see [18]). Some of these genes (e.g., ASFV G ACD 00350 and ASFV G ACD 00600) were expressed (see Supplementary Table S1). The gene designated as pNG4 (in the Ba71V genome) does not seem to have a corresponding gene in the ASFV Georgia_2007/1 genome. Some annotated features within the ASFV Georgia/2007 genome were present within the reads derived from other annotated gene transcripts (e.g., “polyC regions” (nt 14225 to 14237 (within the MGF 110-10-L-MGF110-14L fusion ORF) and nt 15666 to 15682 (within the MGF 110-13Lb ORF) plus a “polyG region” (nt 19993 to 20008, within the ASFV G ACD 00350 ORF)). However, another “polyG region” (nt 17624 to 17632) was expressed at quite high levels (even in the list of total reads not adjusted for the length of the transcript, see Supplementary Table S1). This sequence is present within a very short open reading frame (encoding a 15-residue peptide, MFDLSSILIRGGGPY) that was not annotated previously. The two genes flanking this polyG region were detected at lower levels (see Supplementary Table S1) and, hence, it does not seem possible to account for these reads as read-through from adjacent genes. Three so-called “hypothetical” genes (nt 19411 to 19506, nt 51223 to 51337 and nt 182044 to 182151, see Supplementary Table S1) were detected with many reads. However, the genes annotated as “ASFV G ACD 01760 no indication” (nt 175922 to 176006) and “DP63R no indication” (nt 178506 to 178652) were present within the transcripts from the I177L and MGF 360-16R genes, respectively, and are not listed separately.

Potentially, some sequence reads could be derived from residual ASFV DNA in the samples, even after the DNAseI treatment and purification of the poly(A)+ RNA. However, it appears that the number of such reads should be very low since a number of genes are only included in a small number of reads (<50, see Table 4); this should be compared to >100,000 reads for some of the highly expressed genes (Table 3). The reads derived from genomic DNA should be derived similarly from all the regions of the genome.

We have also “standardized” the number of reads to take into account the size differences between the libraries generated prior to sequencing and the lengths of the different genes. This process had little overall effect on the pattern of highly expressed genes (see Supplementary Table S2). The most highly expressed genes, based on the total reads as listed in Table 3, are also high on the list of the ASFV genes expressed that is shown in Supplementary Table S2, following standardization, e.g., CP312R, CP204L, MGF 100-1L, A151R, K205R and I73R (see also Table 5).

The distribution of RNA transcripts across the genome, derived from the two different strands of the genomic DNA, is shown in Figure 2. Most of the highly expressed transcripts are derived from genes that are well-separated across the genome, and the highly expressed transcripts are copied from each of the strands of the genome. As may be expected, the different strands of the genome are either read in one direction or the other; this prevents the production of mRNAs that are complementary to each other and would form dsRNAs. Two genes, A151R and MGF360-15R, which are highly expressed (see Table 3) are adjacent to each other and transcribed in the same direction (see [17]). However, it is noteworthy that, after the standardization of the number of reads, to take account of the gene length, the apparent level of MGF360-15R expression is less markedly high (Table 5) and does not feature among the most highly expressed genes shown in Figure 2.

The small number of ASFV reads detected in the pigs at 3 dpi (Supplementary Table S1) precludes the accurate quantification of their relative gene expression. However, it is apparent that the few reads that were observed at 3 dpi corresponded to transcripts that were highly expressed at 6 dpi (e.g., see Table 5). It seems most likely that the viral RNA reads observed in the PBMCs at 3 dpi represent the infection of a very small proportion of the cells, consistent with the low levels of ASFV DNA present within the PBMCs at this stage of the infection. It is not possible to differentiate “early” and “late” transcription within the pigs (c.f. in cell culture, [18]) as it cannot be expected that a synchronous state of infection can be achieved. In the RNAseq analysis performed here, the use of alternative transcription start sites has not yet been explored.

## 4. Discussion

The intranasal inoculation of pigs with ASFV is an efficient and consistent means of initiating infection (as seen previously [16]). Each of the inoculated pigs became infected and followed a similar course of disease. Pig 9 was slightly delayed in showing clinical signs, and both pigs 9 and 10 had slightly lower levels of the ASFV derived transcripts within their PBMCs, but it is expected that there will be some differences between animals.

Overall, we have obtained very consistent results among the four pigs infected with the ASFV POL/2015/Podlaskie strain for the expression of each ASFV gene within the PBMCs; this cell population includes the major target cell types (monocytes and macrophages) for this virus. The virus strain used here is very similar to the other closely related genotype II viruses currently circulating in Europe and Asia.

A complete listing of the standardized gene expression data is provided in Supplementary Table S2. In studies described previously by Jaing et al. [19], three pigs were infected (by intranasal instillation) with the ASFV Georgia 2007/1 strain using 10^4^ TCID_50_. The pigs were euthanized on days 7–10 after infection; each of the pigs were shown to have ASFV DNA in the blood from 7 dpi and displayed clinical signs of infection from that time. Total RNA was extracted from whole blood and used for the transcriptomic analysis; only about 0.1% of the reads mapped to the ASFV genome. This is a much lower level than observed here (up to 4.2%, see Table 1); presumably, the much higher proportion of ASFV derived reads that we obtained is largely due to the selection of poly(A)+ mRNAs from the PBMC fraction prior to the sequencing (thus removing many reads derived from other RNAs (e.g., ribosomal RNA). About 1–2 million reads that mapped to the ASFV genome were analyzed here from each of the inoculated pigs at 6 dpi. Another advantage obtained from selecting the poly(A)+ mRNA is that reads derived from residual ASFV DNA should be further diminished beyond that achieved by DNAseI treatment alone, which may not be completely effective [32]. However, some reads may be derived from residual ASFV DNA (there are only short regions of the genome that are not included in any reads), along with other incompletely digested host DNA, e.g., pig mitochondrial DNA. In addition, it is worth noting that <1% of the reads (about 250,000 per sample) do not map to the pig genome nor to ASFV but map to bacterial genome sequences, as determined using Kaiju [31]. It seems likely that this results from bacteria being engulfed by the porcine macrophages and then residual DNA being present within these cells.

A key feature of the data presented here is the close correspondence between the results observed for each of the four pigs. Thus, the most highly expressed ASFV genes observed in one pig were also highly expressed in the other three pigs (Table 3). Indeed, the relative order of expression of the top 20 genes was very similar in each of the four pigs. Furthermore, genes that were expressed in one pig at a low level were also expressed at a low level in the other animals (Table 4). This consistency in the gene expression contrasts with the much more variable results reported previously by Jaing et al. [19]. For example, surprisingly, for nine of the seventeen genes indicated in their study as being highly expressed overall, in one of the three pigs examined, apparently no reads corresponding to these highly expressed genes were detected. Indeed, for the most abundantly expressed transcript overall, encoding MGF-360-15R (an inhibitor of interferon-β induction, [33]), the fragments per kilobase per million mapped fragments (FPKM) values obtained for the three pigs were: 1,190,000; 0 (zero) and 19,979, respectively, showing great variation. This gene was the sixth or seventh most highly expressed gene in each of the four pigs studied here (see Table 3), with the total reads ranging from 23,647 to 49,844 (see Supplementary Table S1). Following the standardization (to account for differences in library size and for the lengths of the genes), this gene was fifteenth in the list of highly expressed genes (Table 5) and, thus, it does not feature among the most highly expressed genes (see Figure 2).

The highly expressed genes of ASFV are mainly located in different regions across the genome and are derived from each strand. There can be a concern that, if the transcription termination signals are not 100% efficient, then read-through into the adjacent genes could affect the apparent level of transcription of the genes adjacent to the highly expressed genes. For the most highly expressed genes that we have analyzed, this could only be an issue for the A151R gene (fourth most highly expressed) and the adjacent MGF360-15R gene (sixth or seventh most highly expressed, see Table 3) since these genes are both transcribed in the same direction. However, after the normalization of the gene reads to take into account the length of the ORF, the expression of the MGF360-15R gene does not appear to be very high (see Table 5 and Figure 2). It seems unlikely that the reads corresponding to the MGF 360-15R gene were greatly affected by the read-through from the higher number of reads derived from the A151R gene unless the transcription termination process was very inefficient. Furthermore, the studies by Cackett et al. [18] indicated a good correlation beyond the transcription measured by RNA-seq (as used here) and the 5′-end cap analysis gene expression sequencing (CAGE-seq). The latter methodology is independent of any read-through transcription. Finally, there was a good correspondence between the genes found to be highly expressed in the pigs, as shown here (see Table 3 and Table 5 and Supplementary Table S1), and those found to be highly expressed during both the early (at 5 h post-infection, prior to DNA replication) and late (at 16 h, after DNA replication) stages of infection in Ba71V-infected Vero cells [18]. Thus, the genes CP312R, CP204L, A151R, K205R and I73R were all found to be highly expressed in both studies. There was also good overall correspondence to the results from Jaing et al. [19] within the pigs, although the higher degree of variability of the signal (as mentioned above) makes for less precise quantification. Many of the ASFV genes are poorly characterized [17], and this applies to the highly expressed genes as well as those expressed at a lower level.

As the functions of the various ASFV genes become known, information about the expression of the genes in different cell types will be important for understanding the biology of the virus. Interestingly, most of the major components of ASF virions (as described previously [34]) are not translated from among the most highly expressed genes. Only the dUTPase component of the virion (from the E165R gene) is produced from among the top 20 expressed transcripts (see Table 3). The genes encoding the major capsid protein (p72 expressed from B646L), the outer envelope protein CD2v (from E402R) plus the two polyproteins pp220 (from CP2475L) and pp65 (from CP530R) do not appear in the list of the top 20 expressed genes. It is noteworthy that the S273R gene, which expresses a protease that processes the two polyproteins (pp220 and pp65) to major components of the virion [34], is expressed at very low levels (Table 4). Clearly, the level of protein expression does not only reflect the level of the mRNA; it will also depend on the stability of the proteins and the translational efficiency of the individual mRNAs. It can be expected that proteins with catalytic functions, e.g., the ASFV protease (S273R), may be required at a lower level than the structural protein precursor substrates.

Overall, it seems that all open reading frames encoding products longer than 60 amino acids (i.e., >180 nt, as annotated in Accession no. FR682468.2) are expressed within the pigs. It could have been possible that some ASFV genes were only expressed in soft ticks as part of its sylvatic cycle, but this does not appear to be the case. However, for the genes that are expressed at very low levels (see Table 3), it is formally possible that these reads are due to the presence of residual ASFV DNA. Clearly, there could be quantitative differences in the pattern of gene expression from the virus when it replicates in the ticks compared to that observed in pigs.

It is apparent that the ASFV must have replicated somewhere in the pigs during the time period from inoculation until after 3 dpi since the virus must have spread to the PBMCs to enable their infection. Following the intranasal infection, it is believed that the primary ASFV replication occurs within the tonsil and regional lymph nodes before being spread, through the lymphatic system and the blood, to other replication sites (e.g., the spleen and liver (see [22]). Further studies should focus on these primary sites of infection and also other sites of subsequent infection. Furthermore, the host responses to the ASFV infection can also be addressed using the PBMCs (as here) and other cells that become infected.

## Figures and Tables

**Table 1:** Description of clinical score system.

Feature	Score	Description
Alertness and recumbency	0	Alert
	1	Depressed/lethargic
	2	Only gets up when touched
	4	Gets up slowly when touched
	6	Remains recumbent when touched
Body condition	0	Normal, full stomach
	1	Empty stomach, sunken flanks
	2	Empty stomach, sunken flanks, loss of muscle mass
	3	Emaciated
Skin	0	Normal
	1	Minimal area of the skin with observed bleeding (<10% of the body)
	2	Moderate area of the skin with observed bleeding (10–25% of the body)
	3	Generalized skin bleeding (>25% of the body)
Joints	0	No joint swelling
	1	Swelling
	4	Severe swelling and lameness
Respiration	0	Normal
	1	Mildly labored
	2	Labored +/− cough
	3	Severely labored
Eyes	0	Normal
	1	Small amount of exudate
	2	Moderate amount of exudate
Gastrointestinal and urinary tracts	0	No diarrhea
	1	Mild diarrhea for less than 24 h
	3	Diarrhea for more than 24 h or vomiting
	4	Bloody diarrhea or blood in urine
Neurology	0	No symptoms
	3	Hesitant, unsteady walk, crossing-over of legs is corrected slowly
	4	Pronounced ataxia
	6	Paralysis or convulsions

This is modified from the system described previously [16] since feed was available ad libitum and rectal temperatures were recorded separately.

**Table 2:** Poly(A)+ mRNA derived sequence reads from PBMCs of pigs infected with ASFV.

Pig Number (Sampling Day)	Number of Input Reads	Number of Reads Uniquely Mapped on Pig Genome	Uniquely Mapped Reads (%) on Pig Genome	Number of Reads Mapped to ASFV Genome	Proportion of ASFV Reads/Total Reads (%)
Pig 9 (0 dpi)	31979021	28863826	90.26	0	0
Pig 9 (3 dpi)	31807126	29207238	91.83	0	0
Pig 9 (6 dpi)	40093403	35472115	88.47	871681	2.17
Pig 10 (0 dpi)	32617753	29668789	90.96	0	0
Pig 10 (3 dpi)	46764030	43095231	92.15	638	0.0014
Pig 10 (6 dpi)	27095889	24288166	89.64	915507	3.38
Pig 11 (0 dpi)	45953582	39771420	86.55	0	0
Pig 11 (3 dpi)	46561959	41696207	89.55	616	0.0013
Pig 11 (6 dpi)	45970436	40372979	87.82	1939287	4.21
Pig 12 (0 dpi)	46326924	37609952	81.18	0	0
Pig 12 (3 dpi)	41406744	36549318	88.27	851	0.0021
Pig 12 (6 dpi)	45915279	41203225	89.74	1945879	4.23

**Table 3:** Total numbers of reads for annotated ASFV genome features that are highly expressed in PBMCs from infected pigs at 6 dpi.

Gene or Feature Name	CDS Start (nt)	CDS End (nt)	Pig9- 6 dpi	Pig10- 6 dpi	Pig11- 6 dpi	Pig12- 6 dpi	^1^ Mean Pigs 11–12
CP312R	128277	129200	66398	78462	138910	144010	141460
CP204L	125783	126367	59137	50204	128247	133890	131069
MGF 100-1L	180479	180904	50566	30183	108077	98680	103379
A151R	49652	50107	45198	45603	96565	89945	93255
K205R	64174	64791	29906	34834	64872	65805	65339
I73R	173088	173306	23887	24501	59167	54809	56988
MGF 360-15R	50346	51215	24275	23647	47350	49844	48597
MGF 110-5L-6L	9490	10107	18740	20840	42820	46048	44434
A240L	48633	49343	18353	15318	41882	45882	43882
MGF 110-7L	10314	10727	20780	20790	43090	42632	42861
E165R	167468	167965	17095	20759	40914	41374	41144
MGF 100-3L	181269	181577	15716	18157	36029	42209	39119
F334L	56956	57960	17153	17920	38103	39044	38574
MGF 110-3L	8239	8613	16201	22633	35842	34045	34944
285L	11042	11326	17175	14716	34762	32868	33815
I215L	174794	175432	16341	14199	32361	32736	32549
MGF 505-3R	35760	36602	13836	11627	25567	30317	27942
DP96R	185339	185629	12107	10550	27516	28141	27829
ASFV G ACD 00600	48000	48152	9902	7709	22675	22563	22619
K196R	65113	65703	9812	14044	22163	22183	22173

^1^: Average number of reads from pigs 11 and 12 at 6 dpi were used to place genes into order. The values given are the total number of sequence reads mapped per annotated region (not standardized for gene length or total number of reads) in the updated ASFV Georgia_2007/1 genome sequence (GenBank Acc. No. FR682468.2). Note that the start and end of the CDS are indicated by their position in the genome independently of the orientation of the gene.

**Table 4:** Total number of reads for annotated ASFV genome features expressed at low levels in PBMC from infected pigs at 6 dpi.

Gene or Feature Name	CDS Start (nt)	CDS End (nt)	Pig9- 6 dpi	Pig10- 6 dpi	Pig11- 6 dpi	Pig12- 6 dpi	^1^ Mean Pigs 11–12	Gene Product Properties (If Known)
ASFV G ACD 01960	187401	187532	141	114	292	254	273	
ASFV G ACD 00190	12456	12581	81	122	265	276	271	
E423R	163803	165074	93	126	240	285	263	
H171R	153250	153765	100	117	264	245	255	
ASFV G ACD 00090	7647	7760	107	150	229	236	233	
L11L	183821	184102	75	79	233	204	219	
B407L	107261	108499	52	119	189	185	187	
O61R	129795	129980	60	126	197	177	187	P12 attachment protein
B117L	106907	107254	66	81	196	165	181	TR containing protein
MGF 505-2R	34093	35673	66	100	197	155	176	
B318L	96276	97232	51	69	152	182	167	Prenyltransferase
E301R	165225	166130	34	74	146	159	153	Proliferating cell nuclear antigen-like protein
EP153R	73808	74284	77	38	139	129	134	C-type lectin-like
ASFV G ACD 01020	92901	93059	47	37	140	88	114	
B119L	95936	96295	38	39	75	109	92	FAD-dependent thiol oxidase
B175L	108527	109054	28	47	82	100	91	Late TF VLTF-2
S273R	147670	148491	20	37	70	89	80	SUMO-1-like protease
E183L	163218	163772	28	46	84	69	77	P54, Virus entry
ASFV G ACD 01870	182604	182741	20	12	85	66	76	
S183L	147058	147609	18	44	55	76	66	
E146L	166164	166604	25	45	69	56	63	PSP
ASFV G ACD 00360	20169	20285	14	14	56	48	52	
ASFV G ACD 00210	13461	13652	18	17	29	51	40	
ASFV G ACD 00240	14570	14680	13	11	26	40	33	
EP84R	71306	71560	7	13	13	26	20	PSP

^1^: The values given are the total number of sequence reads mapped per annotated feature (not standardized for gene length or total number of reads) in the updated ASFV Georgia 2007/1 genome sequence (Acc. No. FR682468.2). Gene product functions (where known) are taken from Dixon et al. [17] and Cackett et al. [18]. PSP = putative signal peptide, TR = transmembrane region.

**Table 5:** Most highly expressed ASFV genes (>180 nt) following standardization.

^1.^ Gene Name	Gene Product Properties (If Known)	Start	End	Pig11- 3 dpi	Pig12- 3 dpi	Pig9- 6 dpi	Pig10- 6 dpi	Pig11- 6 dpi	Pig12- 6 dpi	Mean Pigs 11 and 12
I73R	Tandem repeat sequence	173088	173306	9	13	15236	22260	37512	29371	33441
MGF 100-1L		180479	180904	9	16	16544	14066	35147	27124	31136
CP204L	P32 (P30) phosphoprotein	125783	126367	9	16	14080	17027	30351	26783	28567
A151R	Redox pathway	49652	50107	8	13	13812	19851	29333	23093	26213
CP312R	Immunodominant protein	128277	129200	6	8	10003	16837	20801	18227	19514
MGF 100-3L		181269	181577	4	7	7095	11676	16168	16009	16088
285L		11042	11326	3	8	8409	10263	16917	13520	15219
K205R	In virus factories	64174	64791	3	8	6740	11182	14532	12459	13495
MGF 110-7L		10314	10727	7	3	6996	9970	14420	12059	13239
DP96R		185339	185629	6	4	5805	7205	13114	11336	12225
MGF 110-3L		8239	8613	4	4	6023	11986	13245	10634	11940
E165R	dUTPase	167468	167965	3	5	4783	8273	11378	9725	10551
MGF 110-5L-6L		9490	10107	3	4	4223	6690	9592	8719	9155
A240L	Thymidylate kinase	48633	49343	1	3	3594	4273	8153	7549	7851
MGF 360-15R		50346	51215	2	3	3884	5390	7531	6701	7116
I215L	Ubiquitin conjugating enzyme	174794	175432	2	3	3561	4408	7010	5994	6502
L83L		4878	5123	3	5	2775	2009	6343	5185	5764
MGF 110-4L	Has KDEL-like domain	8927	9301	2	3	2689	4421	6542	4684	5613
A104R	Histone-like	48322	48636	2	4	2532	3481	6119	4943	5531
F334L	Ribonucleotide reductase subunit	56956	57960	2	2	2376	3535	5245	4543	4894
K196R	Thymidine kinase	65113	65703	1	2	2312	4715	5192	4392	4792
MGF 110-2L		7828	8142	2	1	2486	3884	4694	4298	4496

^1^: The total numbers of reads per gene were standardized according to the length of the open reading frame and for the size of the library. Only genes with a coding sequence > 180 nt (encoding > 60 amino acids) were included. The genes are listed according to the mean value for the reads from pigs 11 and 12 at 6 dpi. For comparison, the number of reads generated from the PBMCs at 3 dpi are also indicated for pigs 11 and 12 (for pigs 9 and 10, there were zero (0) or between 0 and 8 reads for each of these genes on this day, respectively). A complete listing of the ASFV genes expressed at 3 and 6 dpi is given in the Supplementary Tables S1 and S2. Gene product properties are from Dixon et al. [17] and Jaing et al. [19].

## Data Availability

The data analyzed in this study are included within the paper and its Supplementary Information (Tables S1 and S2).

## Supplementary Materials

The following are available online at https://www.mdpi.com/article/10.3390/v13112333/s1, Supplementary Table S1: Total numbers of reads for all annotated features in the ASFV genome throughout the course of infection; Supplementary Table S2: Ordered list of standardized number of reads for genes with ORFs > 180 nt expressed in PBMCs at 3 and 6 dpi.
