# Supplementary material for: Identification of African Swine Fever Virus Transcription within Peripheral Blood Mononuclear Cells of Acutely Infected Pigs
Source: Viruses. 2021 Nov 22;13(11):2333. doi: 10.3390/v13112333 (PMC8623482; doi:10.3390/v13112333)
Supplement: Supplementary file 1 [file viruses-13-02333-s001.zip › OlesenetalASFVtranscriptsSupplementaryTable S2word.pdf]

Supplementary Table S2: Ordered list of standardized number of reads for genes with ORFs >180nt expressed in PBMCs at 3 and 6 dpi.

ASFV gene	Pig10-3dpi	Pig11-3dpi	Pig12-3dpi	Pig9-6dpi	Pig10-6dpi	Pig11-6dpi	Pig12-6dpi	Mean (6dpi)
I73R	9	10	14	16349	23959	39774	31684	27942
MGF 100-1L	8	10	17	17753	15140	37267	29261	24855
CP204L	7	9	17	15109	18326	32182	28892	23627
A151R	9	9	14	14822	21366	31102	24912	23051
CP312R	5	6	8	10734	18122	22055	19662	17643
MGF 100-3L	3	4	8	7614	12567	17143	17270	13648
285L	4	3	8	9023	11046	17937	14585	13148
K205R	3	3	8	7232	12036	15408	13441	12029
MGF 110-7L	3	7	3	7507	10731	15290	13009	11634
MGF 110-3L	3	4	4	6463	12901	14044	11472	11220
DP96R	6	7	4	6229	7755	13905	12229	10030
E165R	4	4	5	5132	8904	12064	10491	9148
MGF 110-5L-6L	2	3	4	4532	7201	10170	9405	7827
A240L	3	1	3	3857	4599	8645	8144	6311
MGF 360-15R	2	2	3	4168	5801	7985	7228	6296
I215L	3	2	3	3822	4744	7433	6466	5616
MGF 110-4L	1	2	3	2886	4758	6936	5053	4908
A104R	3	3	4	2717	3747	6488	5332	4571
K196R	1	1	2	2481	5074	5505	4738	4450
L83L	0	3	5	2977	2162	6726	5593	4365
F334L	2	2	3	2549	3805	5562	4901	4204
MGF 110-2L	1	2	1	2667	4180	4977	4636	4115
MGF 505-3R	2	1	2	2452	2944	4450	4538	3596
I10L	2	2	2	2206	2666	5394	4061	3582
MGF 110-8L	1	1	2	2324	2914	4706	3294	3309
DP238L	1	2	2	1708	2254	3794	3537	2823
EP296R	0	0	1	1539	2551	3209	2850	2537
E111R	2	0	2	1534	2276	3402	2774	2496
A224L	1	0	2	1342	2177	3101	2894	2379
C122R	0	1	1	1414	2818	2684	2208	2281
K145R	1	1	2	1332	2030	3111	2501	2243
I8L	1	1	1	1230	1918	3153	2473	2194
EP152R	1	0	2	1567	1752	2904	2269	2123
D205R	1	1	1	1372	1890	2684	2489	2109
I267L	0	1	0	994	1769	2410	2642	1954
I9R	1	2	4	1243	1764	2643	2077	1932
O174L	1	1	1	804	3232	1848	1526	1853
MGF 100-1R	0	0	3	1095	1302	2568	2388	1838
H359L	1	0	1	1075	2116	2312	1828	1833
B354L	1	1	1	789	1727	2842	1505	1716

H339R	1	1	0	752	2020	2235	1749	1689
I7L	0	0	1	957	1729	2221	1769	1669
MGF 360-19Ra	0	0	1	1073	1248	2156	2036	1628
NP419L	0	1	1	856	1790	1973	1852	1618
I243L	1	0	1	904	1528	2019	1975	1607
D339L	1	1	0	886	1682	1992	1854	1604
D345L	0	1	1	918	1682	2110	1641	1588
MGF 110-10-L - MGF110-14L fusion	0	1	1	845	1135	2132	1833	1487
MGF 360-18R	1	1	0	938	1551	1758	1558	1451
MGF 360-4L	1	1	1	885	1092	2066	1667	1427
C129R	1	1	2	804	1433	1867	1460	1391
MGF 360-19Rb	1	0	1	923	864	2048	1725	1390
D205R	1	0	0	753	1412	1698	1300	1291
B602L	0	0	1	559	1361	2006	1199	1281
M448R	0	1	1	672	1470	1424	1385	1238
MGF 360-6L	1	1	0	743	1031	1647	1468	1222
MGF 110-13La	1	2	1	714	1151	1563	1286	1178
MGF 505-1R	0	0	1	717	1061	1457	1471	1176
MGF 300-1L	1	0	0	747	905	1650	1281	1145
MGF 360-1La	1	0	0	656	835	1517	1389	1099
MGF 300-4L	1	0	1	735	891	1426	1241	1073
MGF 360-9L	1	0	1	583	953	1369	1289	1049
A238L	1	0	0	623	892	1274	1404	1048
MGF 360-1Lb	0	1	0	630	817	1308	1357	1028
MGF 300-2R	1	0	1	546	944	1364	1250	1026
DP79L	2	1	0	584	692	1508	1200	996
I329L	0	0	0	501	986	1480	1012	995
F778R	0	0	0	531	984	1144	1087	937
MGF 360-12L	0	0	1	460	889	1001	952	826
MGF 110-1L	0	0	1	536	683	1093	943	814
MGF 360-8L	0	0	0	432	732	994	973	783
C257L	0	0	0	459	720	1061	850	773
A179L	0	0	0	482	629	1028	866	751
MGF 360-21R	0	1	0	427	542	1025	940	734
C315R	0	0	0	441	806	896	748	723
C147L	0	0	1	457	641	925	779	700
MGF 360-14L	0	0	0	501	587	917	788	698
MGF 110-9L	0	0	0	430	551	931	864	694
MGF 110-13Lb	1	0	0	414	599	787	768	642
MGF 360-16R	1	0	1	377	699	754	735	642
CP80R	0	0	1	457	689	802	609	639
MGF 505-6R	0	0	0	411	591	866	687	639
EP424R	0	0	1	371	741	743	696	638
E120R	1	1	1	413	611	777	632	608

MGF 505-11L	0	0	0	283	576	674	742	569
ASFV G ACD 01990	0	0	0	309	524	738	658	557
MGF 505-5R	1	1	0	340	409	745	716	552
H108R	0	0	1	295	402	773	716	547
MGF 505-4R	0	0	1	371	394	701	655	530
H233R	0	0	0	247	532	677	648	526
K78R	0	1	1	298	504	646	626	519
G1211R	0	0	0	256	568	569	618	503
MGF 360-10L	0	0	0	287	474	597	594	488
F1055L	0	0	0	281	497	573	581	483
MGF 505-10R	0	0	0	250	449	595	601	474
NP868R	0	0	0	257	456	559	546	454
MGF 360-13L	0	0	0	264	361	513	467	402
B66L	1	1	0	228	387	549	430	399
B263R	0	0	0	238	397	482	441	390
MGF 505-7R	0	0	0	243	338	499	450	382
C84L	1	1	0	175	471	391	295	333
A859L	0	0	0	191	285	433	364	318
P1192R	0	0	0	174	356	379	364	318
H240R	0	0	0	135	316	377	437	316
D129L	0	0	0	152	312	428	338	308
MGF 110-12L	0	0	0	135	279	445	363	305
MGF 360-11L	0	0	0	179	290	373	365	302
A118R	1	0	0	121	260	570	253	301
C62L	0	0	0	175	352	341	332	300
A137R	0	0	0	129	327	359	286	275
MGF 505-9R	0	0	0	156	234	316	315	255
NP1450L	0	0	0	129	279	298	299	251
DP71L	0	0	1	160	214	334	269	244
X69R	0	0	0	143	257	316	238	238
MGF 360-2L	0	0	0	127	188	322	248	221
ASFV G ACD 00120	0	0	1	119	248	280	221	217
MGF 360-3L	0	0	0	124	194	279	243	210
CP123L	0	0	0	98	244	282	210	208
EP1242L	0	0	0	104	212	234	248	200
EP364R	0	0	0	105	169	265	228	192
R298L	0	0	0	78	126	199	229	158
I196L	0	0	0	59	134	255	135	146
I177L	0	0	0	74	124	206	145	137
F317L	0	0	0	72	127	191	143	133
F165R	0	0	0	45	241	117	114	129
KP177R	0	0	0	73	119	168	135	124
EP402R	0	0	0	69	127	154	137	122
O61R	0	0	0	48	145	155	121	117
QP509L	0	0	0	57	121	161	126	116

H124R	1	0	0	64	104	145	135	112
B125R	0	0	0	66	77	187	105	109
D117L	0	0	0	50	118	139	118	106
I226R	0	0	1	41	166	117	84	102
B385R	0	0	0	43	88	158	108	99
C475L	0	0	0	41	97	116	138	98
E184L	0	0	0	58	73	133	106	92
B646L	0	0	0	41	87	109	100	84
CP2475L	0	0	0	38	84	102	112	84
B169L	0	0	0	48	77	117	94	84
K421R	0	0	0	38	89	101	106	84
E199L	0	0	0	56	65	112	93	82
L11L	1	0	0	40	60	122	91	78
B475L	0	0	0	34	55	105	81	69
E248R	0	0	0	30	73	83	86	68
C717R	0	0	0	28	63	77	62	58
B117L	0	0	0	28	50	83	60	55
Q706L	0	0	0	29	48	71	70	55
H171R	0	0	0	29	48	75	60	53
ASFV G ACD 01980	0	0	0	19	46	77	60	51
C962R	0	0	0	18	47	54	54	43
CP530R	0	0	0	16	40	57	55	42
QP383R	0	0	0	21	40	52	37	37
G1340L	0	0	0	16	36	45	38	34
B962L	0	0	0	17	33	44	36	33
B438L	0	0	0	16	28	36	39	30
EP153R	1	1	0	24	17	42	34	29
B119L	0	0	0	16	23	31	38	27
ASFV G ACD 00210	0	0	0	14	19	22	34	22
E423R	0	0	0	11	21	28	28	22
D1133L	0	0	0	9	20	28	28	21
B175L	0	0	0	8	19	23	24	18
B318L	0	0	0	8	15	23	24	18
E146L	0	0	0	8	22	23	16	17
E301R	0	0	0	6	17	24	22	17
B407L	0	0	0	6	20	22	19	17
E183L	0	0	0	8	18	22	16	16
M1249L	0	0	0	7	14	19	16	14
S183L	0	0	0	5	17	15	17	13
MGF 505-2R	0	0	0	6	13	18	12	13
S273R	0	0	0	4	10	12	14	10
EP84R	0	0	0	4	11	8	13	9
